# How do People in Rural India Perceive Improved Stoves and Clean Fuel? Evidence from Uttar Pradesh and Uttarakhand

**DOI:** 10.3390/ijerph110201341

**Published:** 2014-01-27

**Authors:** Vasundhara Bhojvaid, Marc Jeuland, Abhishek Kar, Jessica J. Lewis, Subhrendu K. Pattanayak, Nithya Ramanathan, Veerabhadran Ramanathan, Ibrahim H. Rehman

**Affiliations:** 1 Department of Sociology, Delhi School of Economics, Delhi University, Delhi 110007, India; 2 Sanford School of Public Policy, Duke University, P.O. Box 90239, Durham, NC 27708, USA; E-Mail: subhrendu.pattanayak@duke.edu; 3 Duke Global Health Institute, Duke University, Durham, NC 27708, USA; 4 The Energy and Resources Institute, New Delhi 110003, India; E-Mails: akar@teri.res.in (A.K.); ihrehman@teri.res.in (I.H.R.); 5 Nicholas School of the Environment, Duke University, Durham, NC 27708, USA; E-Mail: jessica.lewis@duke.edu; 6 Nexleaf Analytics, Los Angeles, CA 90064, USA; E-Mail: nithya@nexleaf.org; 7 Scripps Institution of Oceanography, University of California—San Diego, San Diego, CA 92037, USA; E-Mail: vramanathan@ucsd.edu

**Keywords:** improved cook stoves, air pollution, India

## Abstract

Improved cook stoves (ICS) have been widely touted for their potential to deliver the triple benefits of improved household health and time savings, reduced deforestation and local environmental degradation, and reduced emissions of black carbon, a significant short-term contributor to global climate change. Yet diffusion of ICS technologies among potential users in many low-income settings, including India, remains slow, despite decades of promotion. This paper explores the variation in perceptions of and preferences for ICS in Uttar Pradesh and Uttarakhand, as revealed through a series of semi-structured focus groups and interviews from 11 rural villages or hamlets. We find cautious interest in new ICS technologies, and observe that preferences for ICS are positively related to perceptions of health and time savings. Other respondent and community characteristics, e.g., gender, education, prior experience with clean stoves and institutions promoting similar technologies, and social norms as perceived through the actions of neighbours, also appear important. Though they cannot be considered representative, our results suggest that efforts to increase adoption and use of ICS in rural India will likely require a combination of supply-chain improvements and carefully designed social marketing and promotion campaigns, and possibly incentives, to reduce the up-front cost of stoves.

## 1. Introduction

Over two thirds of all households in India (about 170 million households, or nearly 800 million people) cook on traditional stoves using solid bio-fuels such as wood, agricultural waste, coal and dried cattle manure [1,2]. This practice can adversely impact respiratory health of individuals and local forests and other environmental resources, as well as contribute to climate change [3,4,5,6,7,8]. To confront these negative impacts, a variety of national and local NGO-driven programs have sought to disseminate improved cook stoves (ICS) in India since the 1980s. In the last five years, commercial entities have also begun to enter this sector in India, contributing new technology innovation. Yet prior progress has been slow, and to date these efforts have not successfully reached scale. For example, the largest program implemented by the Indian government, the National Programme on Improved Chulhas (NPIC), which disseminated some 30 million stoves, is widely regarded as a failure [9,10,11]. Furthermore, recent evaluations of several efforts to promote ICS in South Asia suggest that their purported benefits may not have been realized [12,13], though more success has been reported elsewhere, e.g., in Sub-Saharan Africa [14].

Numerous explanations have been offered for the lack of success of previous ICS programs in India [15]. Critics point out that ICS technologies generally remain costly for poor households unless large subsidies are provided, especially with regards to stove cost. The materials needed for traditional mud stoves are free and only require minimal time to construct and maintain. ICS technologies have also often failed to deliver sufficient benefits to justify their adoption by users. The fuel required by ICS technologies may be expensive, both in terms of acquisition and preparation, since some of these stoves (e.g., top loading micro-gasification technologies) generally require higher quality wood chopped into small pieces to deliver the promised gains in efficiency and reduction in smoke levels [16]. The failure of ICS programs has also been tied to an inability to tailor designs to suit user preferences and customs by making it difficult or impossible to cook specific culturally important types of foods [17,18]. In addition, ICS technologies may deliver benefits primarily for women and small children who engage in much of the fuel collection and are most heavily exposed to smoke, and these individuals may have limited decision-making power in poor rural households [19]. Finally, a number of supply-side bottlenecks have likely slowed diffusion of ICS, ranging from constraints on production and insufficient quality control to problems with fuel supply, to a lack of effective social marketing and proper technical and repair support by the promoters of these new technologies [10].

The study described in this paper is not the first to describe household characteristics in the context of stove use in rural areas of developing countries. However, most existing descriptions are based on analysis of general purpose household surveys, not data collection efforts focused on the finer details of household cooking and heating preferences for different technologies. Further, a small portion of this literature focuses on rural India, a high density hotspot for biomass burning (where 30% of global biomass users reside in India [1]) with exceptional diversity, in terms of both population and institutions. To improve understanding of user preferences for and perceptions of ICS, we conducted a series of focus group discussions (FGDs) and semi-structured interviews with more than one hundred participants in several communities located in the states of Uttar Pradesh and Uttarakhand in India in March 2012.

In this paper we discuss the findings from these field interactions with potential users. We focus in particular on two sets of issues: (1) the heterogeneity in user perceptions of the health and environmental effects of traditional stoves, and in their preferences for ICS technologies; and (2) how the perceptions and preferences relate to social stratification, prior experiences, and the influence of neighbors and community leaders. In the next section of this paper we explain our data collection activities. We then proceed to highlight the key results from our field interactions with households, and finally discuss our main findings.

## 2. Methods

Our data collection activities consisted of two types of interactions with households: (1) structured focus groups that followed a specific questionnaire and protocol; and (2) semi-structured interviews with households living in or near communities selected for the focus group discussions. A total of 11 focus groups were conducted during March 2012 in eight different villages or hamlets in CSM Nagar district (Uttar Pradesh) and Nainital district (Uttarakhand) (Table 1). These two locations have very different climates and geographic characteristics: the Uttar Pradesh villages lie in India’s Gangetic Plain, while the sites in Uttarakhand are in the Himalayan foothills. Communities selected for focus groups were chosen to represent locations both with (*n* = 6) and without (*n* = 5) prior experience and knowledge of clean energy projects promoted by The Energy and Resources Institute (TERI) of India. Only one focus group was held in a location with an active TERI ICS project.

**Table 1:** Focus group locations and composition.

Village Name (Hamlet)	State	Focus Group 1	Focus Group 2
Lakhmipur	Uttar Pradesh	Men only (*n* = 10)	Women only (*n* = 10)
Mangrora	Uttar Pradesh	Men only (*n* = 10)	Women only (*n* = 10)
Bijhora	Uttar Pradesh	Men only (*n* = 10)	Women only (*n* = 10)
Kharaitpur ^a^	Uttar Pradesh	Mixed (*n* = 10: 9 women, 1 man)	
Supi (Digarh&Rusali)	Uttarakhand	Women only (*n* = 10)	
Supi (Karholi)	Uttarakhand	Women only (*n* = 10)	
Simail, Gargaon & Darim	Uttarakhand	Women only (*n* = 6)	
Bodibana	Uttarakhand	Mixed (*n* = 7 : 5 men, 2 women)	

Note:^ a^ Site with an active TERI program on ICS.

Each focus group had 6–10 people (total participants = 103), and most were comprised exclusively of women (*n* = 6) or men (*n* = 3). The two other focus groups had mixed groups of men and women; these had five men and two women, and one man and nine women, respectively. Participation in the focus groups was voluntary; upon arrival in a village, facilitators contacted village leaders and gathered people willing and able to join for a community meeting. The village leaders, who did not participate in the focus group discussions, were asked to help assemble a group that was as representative of the social strata in their villages as possible, and to choose ordinary citizens rather than individuals with political standing. All discussions lasted between 1 and 2 hours, and used a survey instrument that contained questions on household demographics and socio-economic status, environmental attitudes, perceptions of different stoves, cooking and fuel use, the health effects of smoke, and a stove decision exercise involving a series of discrete choice tasks.

To explore respondents’ perceptions of the health effects of breathing smoke, we used an elicitation strategy in which respondents allocated ten candies between “safe” and “unsafe” circles to match their views on the health risks incurred by breathing cooking smoke emissions. For example, respondents could place all ten candies in the circle labelled “safe” (*vs.* “unsafe”) if they thought the smoke was perfectly safe (*vs.* definitely not safe) to breathe and had no negative effects on health. To explain the notion of risk, two examples were provided to respondents before eliciting their responses: (a) An equal chance that the smoke would make them sick or not sick (five candies in each circle); and (b) a slightly lower chance that they would get sick than not sick (seven or eight candies in the safe circle).

Respondents in the focus groups also participated in a second elicitation exercise involving choices of stoves with differing characteristics. In this stove decision exercise, all respondents in a group were presented with an identical series of cards depicting three different stoves—two improved and one corresponding to the traditional stove. In Uttar Pradesh, the traditional stove image was a mud *mitti-ka-chula*; in Uttarakhand, a mud *angiti* was shown. From each card, participants were asked to select their preferred stove option. In order to observe responses to a wide variety of stove attributes, two distinct sets of cards with five different attributes were used for this exercise. The first set contained combinations of three different levels of price (1,000; 2,000; and 3,000 Rs.), three levels of fuel requirement (one, three and four units), two levels of smoke emissions (low and high), two methods for fuel loading (top and front), and two capacities (single or double burner). The second contained the same three levels of price and two capacities, as well as two levels of maintenance (low and high), three levels of time requirement (one, two and three units), and three levels of durability (1 year, 3 years, 8 years). Collectively these exercises provide insights and opportunities for extended discussions with respondents about the characteristics of stoves that are most important to them.

Some of these exercises were accompanied by distinct promotional messages that emphasized the benefits of improved cookstoves (ICS) for one of the following: local forests, household health or the global environment. Others specifically asked respondents to imagine how they would be influenced by the adoption behaviour of village leaders or other households in the village.

For the semi-structured interviews, the focus group facilitators and other members of the research team walked with a community guide to visit a range of habitations representing different groups and social strata within a community. Approximately 20 such interviews were held, during which respondents were asked more specific questions about their cooking and fuel collection practices, as well as their experiences with different types of traditional and ICS. Two such respondents also completed a detailed costing exercise related to the time and health costs associated with their fuel collection and cooking practices.

## 3. Results

Our presentation of results is structured around obtaining a more nuanced description of patterns of fuel use and preferences for ICS specific to the sites included in this study. 

**Table 2:** Descriptive Statistics (individual-level questions; *n* = 103).

Variable	Location		Gender		Overall
	Uttar Pradesh	Uttarakhand	Women	Men	
**Employment**					
# agriculture	48	33	53	28	81
#unemployed	8	0	8	0	8
# professional	4	0	3	1	4
# students	3	0	0	3	3
# not seeking work	2	0	1	1	2
# self employed	1	0	1	0	1
**Children**					
% with 1 child	38%	21%	31%	39%	33%
% with 2 children	23%	21%	18%	39%	23%
**Primary cook and decision making**					
% women respondents who are	64%	89%	75%	n.a.	75%
primary cooks					
% men responsible for household decisions	84%	69%	93%	67%	80%
about farming ^b^					
% women responsible for household decisions about cooking and	66%	16%	31%	97%	51%
cooking equipment ^a,b^					
**Religion**					
% Hindu	84%	100%	85%	97%	90%
% Muslim	15%	0	15%	3%	10%
**Caste**					
%Scheduled or Other Backward Castes	67%	0	36%	62%	46%
**Literacy**					
% literate	61%	39%	21%	59%	35%
**Economic status**					
% below poverty line	65%	35%	59%	47%	55%
% households owning a mobile phone	93%	100%	94%	97%	95%
**Electricity**					
Intermittent	38%	100%	58%	53%	56%
No electricity	62%	0	42%	47%	44%
**Household lighting**					
% reliant on kerosene	100%	100%	100%	100%	100%
% reliant on electricity	0%	100%	0%	100%	30%
**Knowledge of ICS**					
% heard of “improved” cook stoves	22%	56%	38%	25%	31%
**Stoves owned**					
% with 1 stove	70%	23%	55%	57%	56%
% with 2 stoves	30%	77%	45%	43%	44%
% with LPG	21%	69%	50%	n.a.	34%
# households with biogas	1	0	0	1	1
# households with kerosene stove	0	1	1	0	1
# households with *sagarh*^c^	0	2	2	0	2
**Stove use**					
% that used traditional mud stoves for	97%	92%	95%	n.a.	95%
cooking activity—Past two weeks ^d^	0%	69%	50%	n.a.	32%
% of owners using LPG stove—					
Past two weeks					
**Fuel use (occasional)** ^4^					
% using firewood	100%	100%	100%	100%	100%
% using dung cakes	67%	0%	54%	n.a.	47%
% using kerosene	38%	38%	59%	n.a.	38%
% using LPG	20%	69%	39%	n.a.	34%
% using leaves and twigs	43%	0%	46%	n.a.	30%
**Fuel collection (not purchased)** ^4^					
% collecting firewood	78%	100%	91%	n.a.	86%
% making dung cakes	82%	n.a.	93%	n.a.	90%
% collecting leaves and twigs	100%	n.a.	69%	n.a.	100%

Notes:^ a^ Many respondents in Uttarakhand said that decision making was not gender specific; ^b^ As reported by women and men respondents, respectively (e.g., 93% of women respondents said that their men were responsible for major household decisions about farming); ^c^ A sagarh is a traditional coal stove; ^d^ Traditional stoves include: mitti ka chulha (mud stove), angithi, and three stone fire; n.a.—Answers to these questions were very limited among men, so that column is left blank. The reported % is for those who report using the fuel.

In particular, we focus below on summarizing observations in the different geographic and climatic zones (Uttar Pradesh and Uttarakhand), as well as the role of gender and social aspects, and prior experience with non- biomass-burning stoves.

### 3.1. Socio-demographics and Household Decision-making

As expected, most respondents were primarily farmers (*n* = 81); the rest were unemployed (*n* = 8), professional (*n* = 4), students (*n* = 3), not seeking work (*n* = 2), or self-employed (*n* = 1) (Table 2). Most had 1 or 2 children (33% and 23%, respectively). Among the women, 75% were the primary cooks in their households. Ninety percent of respondents were Hindu, and only 35% of respondents were literate. Forty-six percent were from government-designated scheduled castes, and 55% were below the poverty line (BPL) or impoverished. These “below poverty line” and “impoverished” classifications are based on the guidelines established by the Planning Commission in India that were in place during the study, and correspond to Rs. 33.33 per capita daily consumption in cities and Rs. 27.2 in rural areas for BPL, and income of less than Rs. 250 per capita per month for impoverished households. Also, fifty-six percent of respondents had intermittent electricity at home (44% had none), while almost all (95%) households owned mobile phones. All households used kerosene for lighting, and 30% also relied on electricity. The majority of respondents (80%) said that men were responsible for household decisions about farming, whereas just over half (51%) claimed that women made decisions about cooking and cooking equipment. Overall, men had greater difficulty and expressed impatience over questions related to domestic cooking practices. As a result, we are unable to report most of the measures of stove and fuel use from the focus groups with men.

### 3.2. Health and Environmental Perceptions

The most pressing village-level environmental problems listed by participants of the focus groups were water quality or scarcity (six groups), deforestation (six), poor sanitation (three), land scarcity (two), and drought (two) (Table 3). Respondents in the mountains (Uttarakhand) tended to be more vocal about environmental problems, particularly deforestation and water quality and scarcity problems. No respondents mentioned air pollution or poor air quality as pressing environmental concerns in response to this open-ended question. However, when asked specifically about air pollution, the most common responses were indoor air pollution or cooking smoke (four groups, all in Uttar Pradesh), and particulate matter or dust outside (two groups, both in Uttarakhand), as well as industrial pollution (1 group in Uttar Pradesh).

**Table 3:** Descriptive Statistics (Group questions; *n* = 11).

Variable	Location (Groups)		Gender (Groups)		Overall (Groups)
	Uttar Pradesh	Uttarakhand	Women	Men	
**Environmental problems perceived**					
as important					
Water scarcity and quality	2	4	5	1	6
Deforestation	3	3	6	0	6
Poor sanitation	3	0	3	0	3
Land scarcity	2	0	2	0	2
Drought	0	2	0	2	2
Air pollution	0	0	0	0	0
**Air pollution related complaints**					
Indoor air pollution or cooking smoke	4	0	3	1	4
Particulate matter or dust outside	0	2	2	0	2
Industrial Pollution	1	0	0	1	1
**Health effected from cooking smoke**					
Watering eyes	5	2	6	1	7
Breathing difficulty	4	2	2	4	6
Cough/cold	4	1	5	0	5
Eyesight effected	3	1	2	2	4
Headache	1	1	2	0	2
Asthma	0	1	1	0	1
Black Lungs	0	1	1	0	1
Tuberculosis	1	0	0	1	1
Skin boils	1	0	1	0	1

**Figure 1:**
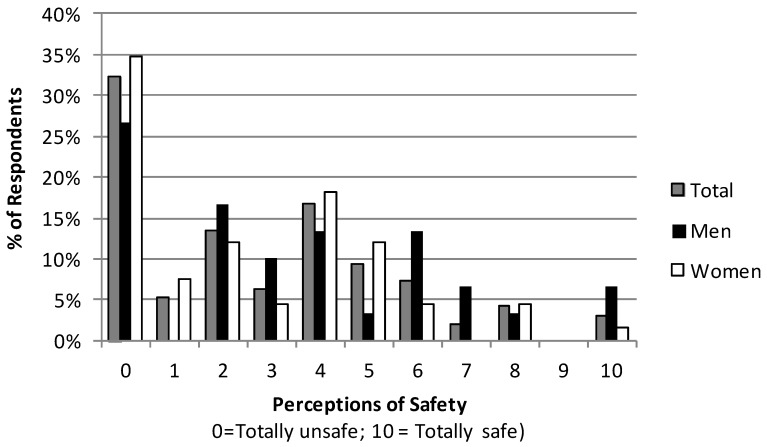
Respondents’ perceptions of health risks from smoke emissions.

The results from the smoke perceptions game show considerable variation across respondents, but suggest that most respondents consider this smoke to be unsafe (Figure 1). Thirty two percent of respondents thought breathing smoke emissions was totally unsafe, and about three-fourths thought the risks that they would become sick were greater than 50%. Groups with men alone thought the risks were somewhat lower, at 27% (definitely making one sick) and 67% (more than 50% likelihood of illness), respectively. Women appeared to think these risks were somewhat higher, and there were no substantial differences in these perceptions by state. Similarly, using a Likert scale, 54% of respondents said that smoke emissions were very dangerous for health, followed by 29% saying they would have serious effects on health. Respondents in these groups listed these health effects to include watering eyes (seven groups), breathing difficulties (six groups), cough or cold (five groups), effects on eyesight (four groups), headache (two groups), asthma, black lungs, tuberculosis and skin boils (one group each).

### 3.3. Cooking Practices (Stoves and Fuel Use)

All focus group respondents had either one (56%) or two (44%) stoves. Thirty four percent of focus group households used LPG stoves, mostly for morning use or making tea; all of the households that reported using LPG in the previous two weeks were in Uttarakhand. Nearly all households relied heavily on traditional mud stoves for the majority of their cooking, most of which (75%) held a single pot. One respondent also used biogas, one had a kerosene stove, and two had *sagarh*s (metal coal) stoves. Households using multiple stoves did so for several reasons: (1) they had large families and multiple stoves made it easier to prepare sufficient amounts of food; (2) they liked the time efficiency of LPG stoves but could not afford the fuel required to use them all the time; and (3) the traditional stoves were more appropriate for certain food preparations, namely slow simmering of milk or cooking breads. The desire for additional cooking options was prevalent in all groups but the specific needs they sought to fulfil with these extra stoves (heat, time savings due to additional cooking surfaces, advantages for specific types of cooking preparations) varied considerably.

In general, respondents from Uttar Pradesh replaced their *mitti-ka-chula* (mud stove) every 3–6 months, though some used the same stove for up to a year. They were relatively easy to build. The *angiti*s used in Uttarakhand, on the other hand, lasted for as long as the house into which they were built, and only required periodic recoating with mud. Ventilated cooking areas were rare except among Muslim households, many of whom cooked outside in the courtyard. The primary reason respondents gave for cooking indoors was a cultural tradition of keeping an enclosed kitchen separated from the rest of the household; this practice was specifically mentioned by Hindu respondents. The lack of an indoor kitchen among Muslim respondents may also stem from differences in socio-economic status, since six of 11 of these were in lower socio-economic categories and may have been unable to construct a separate room for household cooking. Outdoor cooking, on the other hand, offers advantages especially in the hot summer months and in terms of reduced exposure to smoke. Asked about the benefits of traditional stoves, people mentioned their low cost, ease of cooking, satisfaction with the taste of food, and heating benefits in the winter. In response to a question about the main problems with traditional stoves, households mentioned the high levels of smoke emissions, the high fuel requirement, and the time required for cleaning blackened pots and other surfaces. The risk of fire or burns was a constant worry among users of traditional stoves.

All households reported sometimes using firewood as cooking fuel. In Uttar Pradesh, two thirds of households reported using dung cakes and 43% used leaves and twigs, although no households in Uttarakhand used these fuel types. Kerosene was used by 38% of households and LPG by 34%. Though all households used wood, fuel stacking was common, and it was often said in ensuing conversations that many households in Uttar Pradesh used wood sparingly because of high collection and/or monetary cost. The majority of the wood (86%), dung (90%), and leaves and twigs (100%) were collected by households, rather than purchased. Fuel shortage was a problem highlighted by many owners of LPG stoves, which led to payment of higher black market prices for gas cylinders, especially in Uttar Pradesh.

Households in Uttarakhand who participated in a simple costing exercise on fuel collection, cooking time, and cost-of-illness (due to respiratory diseases), were surprised to consider the monetary amounts involved, and particularly those related to time costs. Their responses were generally consistent with similar questions answered in focus groups (where a full costing exercise was not possible): households talked of spending about 3–5 h/week collecting fuel, and women spent about 5 h/day cooking. These time costs, if valued at 10 Rs./h (the reported wage rate alternative for the individuals involved), would be much larger at 1,700 Rs./month than an upper bound on the expected cost-of-illness (175–210 Rs./month) attributable to cooking emissions. In this calculation, the expected cost-of-illness was obtained by multiplying the incidence rate for respiratory disease in the survey areas by household size and an estimate of treatment cost for respiratory illness found in similar developing country settings [16]. This is an upper bound for cost-of-illness because their cooking emissions are only one of many causes of respiratory illness. The value of time, on the other hand, may be an underestimate given that the individuals involved chose not to work at their reported alternative wage rate, but rather chose to spend that time on fuel gathering and cooking.

### 3.4. Knowledge of ICS

Thirty one percent of our respondents (*i.e.*, 26/83) indicated they had heard of ICS that burned biomass, despite the fact that only one of the groups was in a community with a household cookstove promotion program. A second focus group village in Uttar Pradesh had received a “community cookstove” promoted by TERI for cooking the mid-day meal at its school, and a few participants remembered previous government initiatives to promote ICS (probably through the NPIC). In Uttarakhand, many people had heard of “TERI stoves” (a forced draft ICS) via radio broadcasts or word of mouth. The main characteristics of ICS respondents knew about were that they used a fan and required electricity (a feature of the stoves promoted by TERI, as discussed below), required less wood, allowed faster and more efficient cooking, produced less smoke, and were appropriate for a small family. The main concerns respondents expressed about these stoves were the fuel preparation time, the poor taste of food, and maintenance problems.

Experience with ICS, however, was much more limited. Among focus group respondents, only the ten participants from the village in Uttar Pradesh that had an ICS program reported having used an improved stove. Most of those respondents’ stoves were no longer working, and respondents estimated that fewer than 20% were still using the ICS in the village, primarily because of problems maintaining the electrical components required to experience gains in efficiency and reduction of smoke emissions.

The particular technology deployed in this Uttar Pradesh village was a top-loading forced draft stove. Top-loading ICS options are typically most efficient but require addition of fuel through the top of the stove (as opposed to front-loading stoves for which fuel can be added through an opening in the base of the stove). This was judged by many users to be inconvenient because the addition of fuel during cooking typically requires moving the cooking pot. In addition, the forced draft technology (in contrast to natural draft prototypes designed to encourage better passive air flow) uses a battery-powered fan to improve mixing of air and combustion gases in the combustion chamber, which is essential to achieve more complete and cleaner burning, and more efficient heat transfer (*i.e.*, higher efficiency). Unfortunately, this fan and its associated battery system can fail for a variety of reasons, including hot liquid boiling over and damaging the stove circuitry, parts breaking, clogging of the fan, or simple degradation and poor maintenance over time.

The semi-structured interviews conducted near the ICS village—with six households living in different hamlets of that village, as well as four households living in a second nearby village using new ICS forced draft prototypes—confirmed the range of difficulties users experienced with this ICS technology. Respondents also complained about the fuel requirements of the stoves, given that their preferred biomass fuels—dung, twigs, leaves and crop residues—would lead to rapid clogging of the combustion chamber with ash, which greatly diminished stove performance. The limited ability to use all fuel types is perceived as a disadvantage for the ICS, since traditional stoves more readily accommodate fuel mixing. The ICS also required that wood fuel be chopped into small pieces, which takes substantial time and effort. Nonetheless, several respondents in the focus groups and interviews emphasized benefits in terms of time savings from having an additional stove, reduced smoke emissions and heat in the kitchen when it worked, and the advantages stemming from portability of the stove.

### 3.5. Preferences for ICS: Evidence from the Stove Preference Surveys

The results of the stove preference surveys revealed cautious interest in ICS. Across a number of such surveys, respondents indicated some willingness to purchase ICS (Table 4). Depending on the card, 28%–58% respondents said they would be willing to buy one of the two offered improved stoves, though many (particularly among the women) said afterwards that they would need to consult with their spouses before committing. Many respondents (another 33%–54%, depending on the card) liked certain combinations of features but were unwilling to pay for the stoves. These individuals cited as the primary reasons for their resistance a lack of money, the need for subsidies from the government to help them obtain stoves, and general unease about purchasing something that was not readily available in the market and that they did not have experience with. Also, in unstructured discussions, respondents indicated that stove demonstrations and payment plans would be needed for a stove dissemination program to reach them.

In general, respondents had an easier time making choices in the first set of tasks, between attributes of price, smoke emissions, fuel requirement and fuel loading requirement. They had a harder time making choices involving the second set with price, maintenance, time cost and stove durability. The attributes in each of the cards are shown in Table 4: those perceived as most influential in influencing choices in the first set were the number of pots and the fuel requirement (first card), and the smoke emissions, loading and price (second card). With the second set, stove lifespan was by far most important in card 1, while the time cost and price were most important with card 2. All of the features presented were mentioned as important by at least one respondent.

In the focus groups conducted in Uttar Pradesh, respondents indicated that their choices were most influenced by messages related to health and time savings, relative to environmental messages. In Uttarakhand, environmental messages were somewhat more important to respondents, but time savings considerations were most important. Asked what they would do with the extra time saved using more efficient stoves, women typically mentioned household chores and child care, whereas men were more likely to emphasize additional time for farming (due to reduced fuel collection time).

**Table 4:** Summary of results from stove decision exercise.

Description	Set 1 Card 1				Set 1 Card 2			Set 2 Card 1				Set 2 Card 2			
	1-pot ICS	2-pot ICS		Opt Out	1-pot ICS	2-pot ICS	Opt Out	1-pot ICS	2-pot ICS		Opt Out	1-pot ICS	2-pot ICS		Opt Out
Set 1 Attributes															
1a. Price	3,000	2,000		0	1,000	3,000	0								
1b. Smoke	Low	Low		High	Low	High	High								
1c. Fuel requirement	1 unit	3 units		4 units	3 unit	3 units	4 units								
1d. Loading	Top	Front		Front	Front	Front	Front								
Set 2 Attributes															
2a. Price								2,000	3,000		0	1,000	2,000		0
2b. Maintenance								Low	High		Low	High	Low		Low
2c. Time cost								1 unit	2 units		4 units	2 units	1 unit		4 units
2d. Lifespan								8 years	3 years		1 year	3 years	1 year		1 year
% Choosing option	10%	27%		63%	15%	13%	72%	30%	18%		52%	29%	29%		42%
% like but would not buy	13%	40%		n.a.	24%	40%	n.a.	27%	17%		n.a.	16%	17%		n.a.
Attributes of importance in preference															
# of Pots	31%							2%				0%			
Price	11%							9%				22%			
Smoke	15%														
Fuel requirement	24%				12%										
Loading	15%				18%										
Maintenance					37%			15%				12%			
Time cost	4%				11%			22%				44%			
Lifespan					22%			52%				22%			

With regards to hypothetical community adoption behaviour, respondents tended to agree that decisions by others (leaders and other households) not to adopt ICS would likely influence them to not adopt as well. On the other hand, decisions by others to adopt were seen as somewhat less likely to influence their decisions, particularly decisions by village leaders, who were often considered to be in a “different” (higher) social stratum. Participants said the behaviour of their neighbours and relatives would be most influential in affecting their decisions. However, households in the village in Uttar Pradesh with an active ICS program indicated that the village Panchayat’s (local village council) support for ICS was critical to the promotion program in that community. The importance of peer influences in adoption of health-improving technologies has long been considered important in the literature on peer effects and social norms, though the precise nature and mechanism of these influences remains unclear [20,21,22].

## 4. Discussion

The field data collection described in this paper were designed to elicit information on cooking practices among rural households in the states of Uttarakhand and Uttar Pradesh. In particular, these discussions focused on knowledge and experience of improved cookstoves, and perceptions of how they perform relative to traditional cooking options. Here we summarize and discuss the relationships between respondent socio-economic and attitudinal health and environmental variables, and these stove-related beliefs and behaviours. We also reflect on the implications of our research for interventions to promote ICS, acknowledging that our data collection activities were conducted with relatively small samples (that preclude detailed statistical analyses) in two specific environments that are not representative of the overall context where ICS interventions are currently underway.

Through discussions with over one hundred households residing in these locations, the focus group facilitators and study team were able to make two general observations. First, there is considerable heterogeneity in user perceptions of the health and environmental effects of traditional stoves, and in their preferences for ICS technologies. Second, these preferences are related to the social divisions within the surveyed communities, and stated household adoption seems to be influenced by the responses of neighbours experienced with ICS or similar interventions.

Evidence on heterogeneity is apparent from the data presented in the results section of this paper. Specifically, we found that most respondents recognize that there are risks involved in breathing smoke from traditional cooking devices, but that perceptions of the severity of this risk varied a great deal, and were greater among women than men. In addition, views on environmental problems varied with factors such as local context, as revealed by the differences in responses from respondents in Uttarakhand and Uttar Pradesh. These differences reflect variation in priorities as well as differing fuel availability. Households in Uttarakhand were generally much more reliant on firewood collected from local forests than those in Uttar Pradesh, who tended to use a mix of agricultural waste, small twigs, and dung cakes, usually collected from lands near the homestead, supplemented by wood (collected from further away, or purchased in the market to reduce collection time). Finally, different respondents emphasized the importance of different features of hypothetical ICS options that were presented to them. In general, respondents found the capacity, fuel requirement, smoke emissions, loading, price and durability attributes to be important.

We offer a number of comments related to this issue of influences on adoption based on the data collected from these FGDs. First, prior experience with similar interventions in a community had an impact on household responses. Of the 11 focus groups, eight were carried out in communities with prior institutional interventions focused on environmental improvements (three specifically involved ICS technologies and the remaining five focused on clean water provision). We found that this prior experience informed (or perhaps in some cases reflected) the way respondents related and responded to questions about ICS. Respondents in communities more attuned to environmental issues or exposed to environmental interventions through institutions they trusted, tended to respond with greater confidence to features of the stoves that would reduce environmental pressures, such as reduced fuel requirements. Also, the community in Uttar Pradesh that had experience with a specific ICS technology was well versed in the pros and cons of that device. During the group discussion in that village, respondents became much more engaged and interested when presented with attributes of stoves about which they were learning for the first time (e.g., front-loading and multiple burner options).

Second, respondents’ reactions to ICS prices in the stove attribute exercise appeared to have been influenced by prior experience with similar programs. Those in the ICS intervention community (where stoves were provided free of charge) stated that they would pay very little for such stoves (a few agreed that Rs. 2,000 would represent the absolute upper limit of what they would consider, but most suggested much lower prices of 500 Rs. or less). Uttarakhand communities with previous knowledge of ICS distribution efforts (where stove prices varied according to household poverty line status) expected similar arrangements for this ICS program, and questioned the fact that the stove decision exercise lacked discounts for poor households. In communities with limited prior experience with such technologies, respondents tended to make comparisons of the suggested ICS prices with those of other goods and investments that the households might make instead. These sorts of responses show the effects that prior interventions can have on the way that communities assess the value of the ICS. They also suggest that substantial price discounts might be required to achieve widespread stove adoption.

Third, the heterogeneity in preferences and beliefs about ICS can at least partly be explained by household socio-economic characteristics such as education and social status. For instance, women who claimed to occasionally use the ICS stressed that they found the stove technology easy to use. They also suggested in follow-up questions that those who had trouble using the ICS were less worldly and less travelled than they were. Similarly, these women argued that villagers complaining about the taste of food cooked on the ICS tended to be older and male (and that younger men who had migrated outside of the village unit had no such problems). Also, in non-stove intervention communities, privileged households were much more likely to have engaged with other cooking technologies (mostly LPG stoves). They were therefore more amenable to the idea of paying for an ICS device, both because of the possibility of efficiency and health gains, and because it would also be viewed as a status symbol. This group could more readily envision a stove that functioned better than the traditional ones on which they relied. Thus, the group of younger, more educated, wealthy, and connected villagers seemed more open to experimenting with the new cooking technology and to the possibility that it could lead to positive changes in their everyday lives. Such people might be early adopters, although their higher use of other clean stoves means that they might find less use for ICS than others. Finally, differences in openness to experimentation and learning about stove features appeared greatest in communities with limited prior exposure to environmental interventions and institutions.

Fourth, gender significantly affected the responses registered in interactions with respondents, an issue that has been highlighted in other research on the demand for ICS [12,19]. As mentioned above, women participants, most of whom (75%) were the primary household cooks, seemed more aware of the ill effects of smoke emissions produced during cooking. While many men acknowledged these health risks as well (as demonstrated in the health risk perceptions exercise), they did not see these risks as critical issues affecting their daily lives. Male respondents also often found questions related to cooking practices and fuel use irksome. They objected to many of the questions that were included in the focus group script, suggesting that facilitators should ask their wives about such details. At the same time, questions related to stated willingness to pay for ICS were difficult to answer for most women respondents, perhaps because only a third of women claimed they were responsible for household decisions related to stoves and cooking (interestingly, virtually all male respondents (97%) stated that women had this responsibility). Women typically expressed a need to consult their husbands or other male decision makers in their household before making financial commitments. The few women who were themselves heads of households (widows or husbands had migrated to urban centres) were able to more confidently and deliberately consider the cost of the ICS.

There was also a difference in the way that men and women perceived health and fuel issues in the communities sampled in Uttar Pradesh and Uttarakhand. Though differences across these locations deserve additional study, we consider that this divergence may be at least partly related to differences in gender roles and status in the two regions. We found that women’s mobility was highly restricted in Uttar Pradesh relative to Uttarakhand. Many respondents participating in the semi-structured interviews in those communities noted that the mobility of women was limited due to caste differentiation and religious norms. This in turn meant that adult women in Uttar Pradesh were rarely involved in the collection of fuel wood, which was instead a job assigned to men or children. In Uttarakhand, on the other hand, where women’s mobility was less restricted, women and men alike frequently travelled much further to collect fuel. Thus, the time savings from reduced fuel collection needs was much more salient for men in Uttar Pradesh than for women, whereas both men and women in Uttarakhand judged such savings to be important. Indeed, because women tended to have lower decision-making power in our study households, their concerns over emissions of smoke would likewise have reduced importance in purchasing decisions relative to shared benefits such as reduced fuel needs.

Fifth, we found that despite the variations in context and heterogeneity of preferences within communities, messages related to time savings due to more efficient cooking and lower fuel requirements (and hence reduced fuel collection needs) seemed to resonate most with respondents. The way that particular components of time savings were viewed again varied, however, and the range of these responses is consistent with the heterogeneity in benefits from time savings associated with changing cooking practices [16]. For example, women tended to express a much stronger preference for a double pot ICS than men, for two reasons: time saved while cooking due to additional cooking capacity, and time saved in the collection of fuel wood due to increased efficiency of the ICS. These preferences are consistent with the opinions expressed in the community with an active stove promotion program and those for users of alternative technologies such as LPG. Men on the other hand placed more weight on the time saved in the collection of fuel wood (especially in Uttar Pradesh), or on the fact that the ICS would reduce pressure on local forests (especially in Uttarakhand). This also explains why environmental messages on forest protection were judged to be somewhat important by respondents in Uttarakhand: these respondents consistently voiced concerns over the reduction in forest density and what they interpreted to be a corresponding depletion of water sources. They also made connections between human activity and a perceived decrease in fuelwood availability. In the stove decision exercise, men were much more likely than women to make the switch to single pot options when the price of the double-burner ICS was higher.

Despite the salience of the time savings messages for households, it should be noted that the relative cost of cooking with traditional stoves may not be fully known to households. This was noted during the costing exercise carried out with respondents in Uttarakhand, in which the wage rate of household members involved in cooking and fuel collection, and the time spent on these activities, were multiplied together to derive a rough estimate of their effective price. Respondents participating in this exercise had clearly not attempted such calculations before, and they noted in debriefing questions that they had never fully internalized these recurring costs. To participants completing this costing exercise, the idea of an ICS offering potential savings from reduced time spent cooking and collecting fuel then became even more salient, and these respondents were better able to compare the costs of such a stove (both in terms of upfront costs and recurring maintenance) with the benefits that might accrue to them over time. The increased awareness this costing exercise evoked offers hope that information and awareness-raising activities might be effective tools for ICS promotion.

Finally, based on responses in the focus groups and other interviews, it seems likely that the adoption decision would be heavily influenced by the marketing of the intervention and the dynamics of local community adoption [12]. All respondents said that they would need to see (and possibly use) the ICS in question before they made any sort of an investment. Further, they generally agreed that the views of leaders in the community regarding ICS (*Pradhans* or other influential persons), while significant, would be less critical to their adoption decisions than those of their immediate neighbours and kin. In fact, most respondents in Uttar Pradesh said that they had only limited exposure to village leaders and therefore would have little opportunity to see or hear about the ICS from such people, who were in a different class from them. Similarly, in Uttarakhand, where villages tended to be highly segregated spatially, most members of the village ventured only rarely to the *Pradhan’s* hamlet. Thus, promotion of ICS in these communities might require intensive social marketing efforts and free trials rather than simply relying solely on approval and adoption of opinion leaders such as the *Pradhan* [23].

## 5. Conclusions and Policy Implications

In conclusion, while our findings are based on a limited set of group discussions and interviews (compared to a large survey) in rural areas of northern India, we believe we confirm existing hypotheses and generate new insights on the nature of the challenge of achieving widespread adoption of improved cookstoves [15]. In particular, promotional messages should emphasize the high costs of traditional stove use, and those conducting ICS interventions should understand that lack of experience and exposure to such technologies can impede adoption. Given current efforts to increase promotion of ICS technologies in India and other less-developed countries, we believe that issues related to stove promotion and marketing, stove pricing and subsidies, and the nature of peer effects and how they relate to the visibility of stoves deserve additional study and rigorous testing. In particular, we feel that small-scale experimental approaches testing different technologies and promotional programs across different settings is required before undertaking broad ICS dissemination campaigns. Only then will a nuanced picture of the broader context of supply and demand of such technologies and the heterogeneity of household knowledge and preferences emerge.
